# Reporting Nasogastric Tube ‘in the Stomach’ Is Not Enough: Full Intragastric Positioning Matters

**DOI:** 10.5334/jbsr.4131

**Published:** 2025-11-07

**Authors:** Thalinne Schueremans, Nico Hustings

**Affiliations:** 1 Department of Radiology, UZ Leuven Gasthuisberg, Leuven, Belgium; 2 Department of Radiology, SFZ, Heusden-Zolder, Belgium

**Keywords:** tubes, aspiration, nasogastric tube

## Abstract

*Teaching point:* Even when the nasogastric tube (NGT) tip appears intragastric on chest radiography, malposition of proximal side holes above the gastro-oesophageal junction may result in clinically significant aspiration.

## Case History

A 67-year-old woman was admitted to the ICU due to haemorrhage after robot-assisted left partial nephrectomy. Her surgical history included low anterior resection, gastric bypass, and pacemaker implantation.

**Figure 1:**
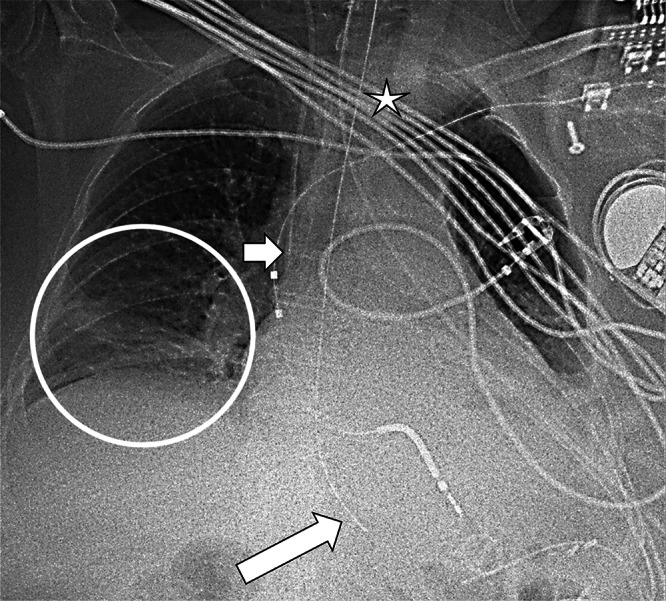
Chest radiography shows multiple supportive devices (star = endotracheal tube; short arrow = jugular central venous catheter; long arrow = nasogastric tube, NGT) and a lung consolidation in the right lower lobe (circle). The tip of the NGT projects under the diaphragm.

During her ICU stay, the patient remained under general anaesthesia with multiple supportive devices in situ, as shown on chest radiography (Figure 1). An endotracheal tube was correctly positioned with its tip projecting above the carina (*star*). A right-sided internal jugular central venous catheter was in place, with its tip projecting at the atriocaval junction (*short arrow*). A nasogastric tube (NGT) was present with the tip projecting just below the left hemidiaphragm (*long arrow*). Additionally, a lung parenchymal consolidation was noted in the right lower lobe (*circle*).

**Figure 2:**
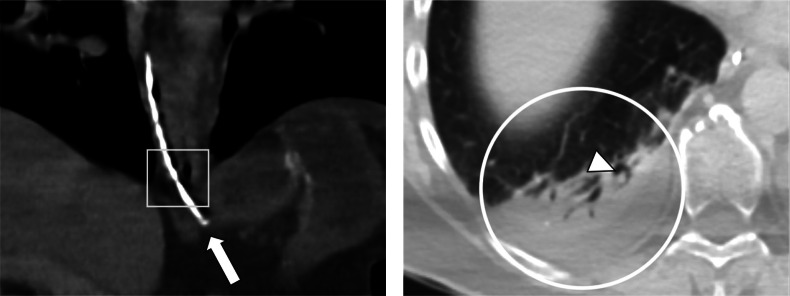
**(Left picture)** CT confirms the tip of the NGT inside the gastric cardia (arrow), only slightly distal to the lower oesophageal sphincter (square). **(Right picture)** Using lung windows, a right posterobasal consolidation and small adjacent pleural effusion (circle) were observed with intraluminal soft-tissue densities (arrowhead) in the supplying airways. This suggests aspiration as the cause.

Figure 2 shows images of computed tomography (CT) of the chest confirming the NGT tip was located within the gastric cardia (*arrow*), though only slightly distal to the lower oesophageal sphincter (*square box*). With appropriate window settings, a right posterobasal consolidation accompanied by a small adjacent pleural effusion (*circle*) was observed. Intraluminal soft-tissue densities (*arrowhead*) were noted in the airways supplying the affected lung segment, suggesting aspiration as the cause.

## Comment

**Figure 3:**
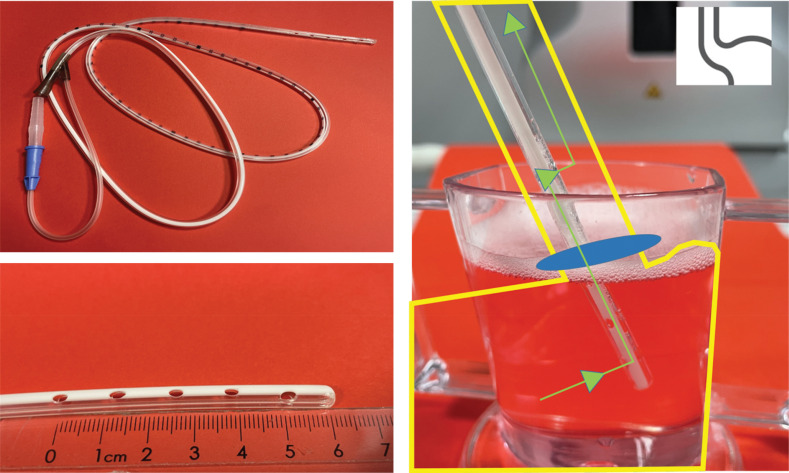
**(Left pictures)** show the functional design of the NGT with several side holes located several centimetres proximally to the tip. **(Right picture)** demonstrates gastric reflux (green arrow) entering the NGT through a distal side hole, flowing retrograde across the sphincter (blue circle), and re-entering the oesophagus via a proximal side hole and thus potentially reaching the airways. (yellow figure: top = oesophagus; bottom = stomach).

A common misconception is that reporting placement of a tube ‘in the stomach’ on radiological imaging is sufficient. However, this overlooks the functional design of the NGT, which includes several side holes located several centimetres proximally to the tip as shown in Figure 3 (left pictures).

Both the tip and all side holes must be fully positioned within the gastric lumen, distal to the gastro-oesophageal junction. If any opening remains in the oesophagus, gastric contents can reflux into the upper airway, increasing aspiration risk. This mechanism is visualized in Figure 3 (illustration on the right), the oesophagus (top), stomach (bottom), and in between the lower oesophageal sphincter (*blue circle*). A green arrow demonstrates reflux: gastric contents entering through a distal side hole, flowing retrograde through the tube across the sphincter, and re-entering the oesophagus via a proximal side hole and thus potentially reaching the airways.

Current guidelines recommend that the tip be placed at least 10 cm distal to the gastro-oesophageal junction [1]. The traditional NEX (nose–earlobe–xiphoid) method, though commonly used, may underestimate the required length: a safety margin of an extra 10 cm is now advised.

Radiographic confirmation is particularly important in high-risk patients (e.g. sedation). On chest radiography, the NGT should follow the expected path: descending centrally in the thorax without kinking or tracking the airways, crossing the diaphragm at the oesophageal hiatus (typically left infradiaphragmatic), and terminating at least 10 cm distal to the level of the hiatus.

## References

[r1] Yuliati M, Ganefianty A, Afianty N, Kurniawan T. A clinical review of estimating the accuracy of nasogastric tube insertion depth. J Holist Nurs Science. 2023:10(2);8811. 10.31603/nursing.v10i2.8811.

